# Extracellular Vesicles in the Synovial Joint: Is there a Role in the Pathophysiology of Osteoarthritis?

**DOI:** 10.5704/MOJ.1903.012

**Published:** 2019-03

**Authors:** A Esa, KD Connolly, R Williams, CW Archer

**Affiliations:** School of Bioscience, Cardiff University, Cardiff, United Kingdom; * Swansea University Medical School, Swansea University, Swansea, United Kingdom; ** School of Chemistry, Cardiff University, Cardiff, United Kingdom

**Keywords:** articular cartilage, osteoarthritis, extracellular vesicles, biomarkers, therapeutics

## Abstract

The role of extracellular vesicles (EV) in osteoarthritis has become the focus of much research. These vesicles were isolated from several cell types found in synovial joint including chondrocytes and synovium. As articular cartilage is an avascular tissue surrounded by synovial fluid, it is believed that EV might play a crucial role in the homeostasis of cartilage and also could hold key information in the pathogenesis of osteoarthritis. This is thought to be due to activation of pro-inflammatory factors leading to a catabolic state and degradation of cartilage. In addition, due to the nature of articular cartilage lacking neuronal innervation, knowledge of EV can contribute to identification of novel biomarkers in this debilitating condition. This can be either directly isolated from aspirate of synovial fluid or from peripheral blood. Finally, EVs are known to shuttle important signalling molecules which can be utilised as unique modality in transferring therapeutic compounds in a cell free manner.

## The synovial joint and articular cartilage

Articular cartilage is composed of an extensive extracellular matrix (ECM) in which a single cell type, the chondrocyte, is embedded. Chondrocytes are unique cells that are approximately 10 μm in diameter and are sparsely distributed within the ECM, accounting for only 2-5% of the total tissue volume^1-2^. Chondrocytes have many organelles including the endoplasmic reticulum and Golgi apparatus, which are required to produce large quantities of essential matrix components. In addition, these cells have intracellular secretory vesicles, lysosomes and intra-cytoplasmic filaments all contributing to maintaining joint homeostasis. The most common pathological condition affecting synovial joint is osteoarthritis.

## Osteoarthritis (OA): A brief overview

Worldwide, osteoarthritis (OA) affects one in three people between the ages of 18-643, often leading to a debilitating lifestyle for sufferers of the condition. Unfortunately, this number is on the rise with an increasing aging population and related conditions, such as obesity, increasing the numbers of those being diagnosed. Osteoarthritis was generally considered to be a ‘wear and tear’ disease, predominantly affecting articular cartilage and subchondral bone, maintained with painkillers, non-steroidal anti-inflammatories and, in the late stage, joint replacement^4^. However, this somewhat simplistic concept is being challenged, resulting in a paradigm shift of our view of the pathophysiology of OA. Osteoarthritis is now recognised as a complex syndrome effecting multiple tissues within the synovial joint and involving many complicated homeostatic pathways^5^. The discovery of stem-cell like cells and MSC-OA cells within the cartilage has also led to exciting research that has extended our knowledge of the joint and thus cartilage tissue repair^6,7^.

In the articular cartilage of healthy adults, there is an equilibrium between catabolism and anabolism to ensure the ECM is maintained both structurally and functionally throughout the life-span of an individual^8^. During the progression of OA, chondrocytes and other tissues become activated due to exposure to abnormal environmental insults and the homeostatic balance is altered. Pro-inflammatory factors are released and catabolic activation begins resulting in a nett degradation of cartilage ECM. The mechanisms of cartilage degradation are well understood. Early OA is defined by aggrecanases (ADAMTS)-induced proteoglycan loss, whereas late-stage OA is characterised by subsequent degradation of the collagen network. This latter stage is induced by various matrix metalloproteinases (MMPs), which cause irreversible collagen depletion and chondrocyte apoptosis, ultimately compromising the tissue’s functional ability of mechanical force dissipation, an irreversible process whereby the cartilage cannot repair^9-11^.

Research into OA is focused on ways that we can halt the progression of the disease and enhance the natural repair mechanism of the tissues, whether this is by using drugs to prevent catabolic pathways, implanting bio-scaffolds seeding with cells, potentially cartilage-MSCs, or using genetic techniques to ‘change’ cell function, we still have a long way to go before these treatments reach the patient. It is clear however, that the biology of the chondrocyte is an essential avenue of research that needs to be investigated in order that we might learn how the components of the cell function and how we can use these findings to develop preventative or reparative treatment regimes.

## Extracellular vesicles (EVs)

Extracellular vesicles (EVs) are a group of submicron, membrane-derived vesicles secreted by all human cell types^12-13^. The term “extracellular vesicles” is largely used to describe exosomes, microvesicles and apoptotic vesicles which differ in size, biogenesis and biomolecular composition, although there is no clear cut test to classify each sub-group of vesicles.

Briefly, exosomes are generally small in diameter (<150 nm) and are produced as part of the endocytic pathway from fusion of multivesicular bodies with the plasma membrane. Hence, exosomes typically contain endocytic proteins such as Rab proteins, Alix, TSG101 and Lamp 2^14^. In contrast, microvesicles arise from a disruption of both the phospholipid asymmetry and the actin cytoskeleton of the plasma membrane resulting in budding of vesicles directly into the extracellular space^15^. Consequently, microvesicles are generally larger in diameter compared with exosomes (100 nm - 1μm), are more heterogeneous and bear many surface characteristics of the cell of origin. Apoptotic vesicles are also classified as EVs, formed by plasma membrane blebbing of cells undergoing apoptosis. These are the largest, ranging from 100-5000nm in size. The EVs are thought to harbour specific tetraspanins including CD9, CD63 and CD81, as well as biomolecules from the cell of origin and integrins – receptor proteins that can bind and respond to the ECM^15-16^. Functional lipids and nucleic acids, predominantly mRNAs and microRNAs (miRs) can also be present alongside cellular components such as DNA and RNA which can be delivered to and affect the function of cells. The presence of these biological cargo has resulted in research into the role of EVs in various physiological processes, including haemostasis, angiogenesis and immunity^17-19^. More recently focus of current EV research has concentrated on their roles in disease as alterations in quantity and content of EVs have been shown to have detrimental effects in numerous types of cancers, cardiovascular and autoimmune diseases^20-22^.

## EVs and OA

EVs have been detected in a variety of biological fluids, most commonly plasma, urine and conditioned culture media but relevant to our discussion here is that EVs are found in the synovial fluid that fills the joint space^23,24^. The isolation of EVs from synovial fluid is fairly recent and therefore, less well characterised than other biological fluids^25^. Consequently, the majority of evidence for a role for EVs in the synovial joint comes from *in vitro* data. Data has indicated there is little difference between OA patients and healthy subjects in the size and concentration of EVs isolated from synovial fluid^26^ suggesting pathological differences may reside in the molecular composition of EVs. Indeed, EVs isolated from synovial fibroblasts treated with the pro-inflammatory cytokine IL-1β (to mimic conditions of OA) were able to induce MMP-13 and aggrecan expression in articular chondrocytes isolated from healthy synovial joints, suggesting *in vitro* this would lead to tissue degeneration^30^. It is well known in OA, that the synthesis and activation of proteolytic enzymes such as MMPs and aggrecanases is induced through the action of pro-inflammatory cytokines such as tumour necrosis factor (TNF)-α and interleukin (IL)-1β. Activation of these enzymes causes degradation of the cartilage ECM leading to progressive cartilage damage. This in turn induces further TNF-α and IL-1β generation via an autocrine mechanism, and the production of other pro-inflammatory cytokines such as IL-6 and IL-8, causing a vicious cycle of inflammatory-driven degradation^27-29^. This preliminary study demonstrates that EVs produced by both synovial fibroblasts and chondrocytes under OA-like conditions upregulate the release of pro-inflammatory cytokine cascades, including MMP-13, creating a “positive-feedback loop” that drives inflammation within the joint and ultimately leads to the damage of articular cartilage and a loss of structural integrity^26^. Already it is evident that EVs are playing a role in the destructive cascade that exacerbates OA.

## EVs derived from mesenchymal stem cells (MSCs)

A surge in research from many groups has focused on the possibility of mesenchymal stem cells (MSCs) for use in tissue repair, including cartilage. This is due to MSCs multipotent property in that they can differentiate into the mesenchymal lineages; bone, cartilage and adipose tissue^31,32^. Several approaches were adopted in cartilage tissue engineering including injection of MSCs directly into the defect and placement of periosteal flap or lately embedding MSCs in a synthetic matrix plug to fill the defect^4,33^. Unfortunately, even with the discovery of a rare cohort of articular cartilage stem cells in both OA and healthy tissue, none of these current treatment therapies elicit long term repair^6,34-36^.

It was recently discovered that EVs can also be derived from MSCs and potentially possess extensive therapeutic use in wide array of human diseases. A handful of studies have shown positive outcomes in using MSC-derived EVs to promote cartilage repair and protect against OA-induced cartilage degeneration^37-40^. The method of MSC-EV action is still not entirely clear but it seems to be due to the release of paracrine factors. Briefly, the mechanism by which EVs derived from MSCs function is predominantly based on the transfer of miRs; therapeutic effects have been demonstrated in reduced cardiac fibrosis following myocardial infarction and encourages functional recovery after stroke by means of neural plasticity^41,42^.

## EVs and MiRNAs

One of the widely known facts about EVs is that they contain miRs. MiRs are a novel group of non-coding single stranded RNA of 19-24 nucleotides. They have the ability to modulate a large proportion of the genome post-transcriptionally as they can bind to the 3’ untranslated region (UTR), or sometimes the 5- UTRs, of the multiple mRNA targets. So, in essence, one specific miRNA can inhibit the translation of multiple genes. This mode of action has established miRs role in normal cellular homeostasis as crucial, and its dysregulation is therefore associated with a wide range of pathological conditions^43^. MiRs have been elucidated in animal models of RA; considered the inflammatory joint disease, and it is becoming evident that although the aetiology of OA is different, similar miRs to those present in RA have been discovered. More than one study has demonstrated a decrease in the miRNA-146a in human synovial fibroblasts and in animal models of OA^44,45^. Another researched miR is miRNA-140; it is highly expressed in chondrocytes and Hong *et al* have demonstrated its importance in cartilage homeostasis^46^. Following on from this study, miRNA-140 has been shown to be lower in chondrocytes from OA patients^47,48^. MiRs can also be found in MSC-EVs as mentioned previously in this review. In *in vivo* rat OA models intra articular injections of MSC exosomes partly prevented OA damage. Over expression of miRNA-140-5p in the MSC exosome increased this effect^40^. Research demonstrates that miRNAs play a vital role in maintaining a healthy joint and their imbalance could potentially lead to the degradation and eventual destruction of the diarthrodial joint. On the flip side of this, if the miRs that contribute to the signalling cascades involved in tissue degeneration, targeting of these miRs has the potential of de-activating pro-inflammatory pathways to slow down or even prevent disease.

## Ev therapeutics

### Biomarkers

Given the prevalence of EVs in the synovial fluid derived from synovial fibroblasts and chondrocytes in both healthy and normal joints it would suggest that EVs have the potential to function both as an invasive and non-invasive diagnostic tool. Unfortunately, few studies have profiled EVs from synovial fluid. This can be explained in part due to the invasiveness entailed in isolating such biofluid. If research were to concentrate on the area of OA then there is little reason why using the synovial fluid or even the blood plasma EVs as biomarkers of disease state is not feasible. This would, however, rely on the EVs being comprehensively profiled. To date a handful of studies have isolated and profiled EVs with particular concentration on the miRs. Withrow *et al*, recently found that EVs isolated from the synovial fluid of patients with OA were enriched in miR-200c compared with healthy controls^26^. Upregulation of this miRs may accelerate articular cartilage degradation; EV-associated miR-200c may therefore prove a useful biomarker in the early stages of OA development. Kolhe *et al* observed gender specific differences in the miRs content of EVs from OA compared to non-OA subjects^49^. Here, several miRs involved in oestrogen production, signalling and inflammatory cascades were differentially expressed in EVs from females with OA. A link that may relate to the increased prevalence of OA in post-menopausal females where oestrogen production is reduced and inflammation is enhanced.

Answering some of these questions, and ensuring healthy tissues are included in the studies, could even provide data that would allow us to predict whether an individual could be pre disposed to degenerative or an autoimmune disease that would lead to cartilage disorders.

## Tissue repair/joint homeostasis

EVs could potentially allow tissue repair and long-term joint homeostasis if we were to understand their mechanisms of action and communication with surrounding tissues. EVs are endogenous vehicles, making them superior to liposomes as therapeutic agents as they are much less likely to be degraded and therefore protect and enhance the stability of their cargo. EVs may also be strategically loaded with exogenous treatments (e.g. pharmacological agents, proteins, nucleic acids).

**Fig. 1:**
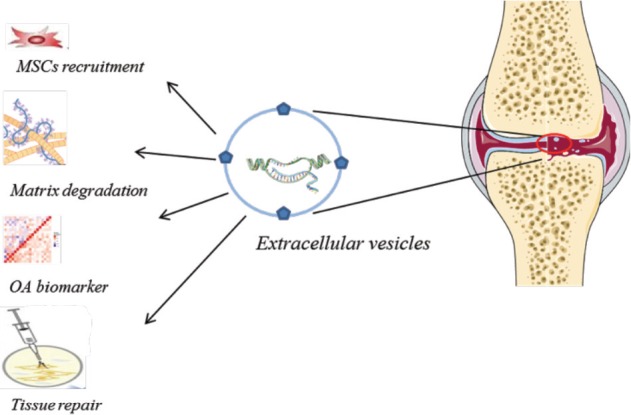
Extracellular vesicles (EVs) in osteoarthritis. EVs released from synovial joint play several roles. EVs are involved in MSCs recruitment and tissue repair, tissue destruction and matrix degradation. EVs are thought to be potential tool for tissue repair and can be utilised as tissue biomarkers.

As EVs play a role in regulating cell recruitment, proliferation and differentiation, therefore having the knowledge of how the EVs are involved in these mechanisms would allow for the potential to control and induce tissue regeneration. Indeed, MSC-EVs exerted an immunosuppressive and anti-inflammatory effect in one study investigating MSCs as modulators of joint homeostasis^37^. Cell repair therapies for cartilage defects at present are concentrating on seeding various biological scaffolds with MCS and promising results have been recorded. However, it is likely that MSC-EVs are involved in this cell recruitment, differentiation and repair response. If we discover the mechanisms by which the MSC-EVs are involved in the tissue repair process then MSCs could be replaced with MSC-derived EVs. This procedure could be enhanced with the possibility of activating or de-activating certain proteins/miRs to increase repair. As such, a cell-free treatment would reduce some clinical regulations and therefore reduce the time from lab to the clinic for novel therapeutics (Fig. 1).

## EV preparation and selection

Whilst the application of EVs in cartilage repair therapies is promising *in vitro*, outcomes are often dependent on parameters such as the quality and reproducibility of cell type, pre-analytical processing of EVs and EV characterisation. Although many studies have isolated EVs from biological fluids, including that of synovial fluid, there is no standard method of which to do this. As the EV isolation procedure determines the EV-mediated functional effects then this process, if not standardised, will introduce variations in data and results. Purifying EVs using ultracentrifugation and density gradient flotation should provide a fraction that is not contaminated with several EV subtypes and non-vesicular particles. For example, MSC derived EVs are dynamic, much like their cell of origin and it has been shown that their contents can be altered dependent on the tissue type cultured^50^. We are already aware that MSCs require bioengineering to be able to differentiate into multiple lineages as adipocytes, chondrocytes and osteoblasts^51^. Therefore, contextualising the use of MSC-derived EVs in cartilage regeneration may be achieved by the content of EVs altering the microenvironment. Delivering the EVs in a biomaterial scaffold could achieve the desired outcome as although EVs could be injected into the joint space, this would be actively cleared. If EVs are to be used in clinical applications then this is one area where research needs to be targeted.

## Conclusion and future work

Recent advances in our understanding of the complexity of OA are helping to identify new mechanisms of disease development, and subsequently, potential targets for therapy, some of which are already reaching the clinics. Unfortunately, due to the nature of the cartilage, repairing defects within the joint have presented with little success, and maybe we need to concentrate on reducing disease progression. There is accumulating evidence for a role for EVs from synovial fluid, fibroblasts and chondrocytes, as well as in the circulating blood plasma, that play an important role in maintaining a healthy fully-functioning joint. It is becoming apparent that joint homeostasis involves the contents or signalling cascades that the EVs are involved in, and during the development and progression of OA these mechanisms become unbalanced. The exact mechanism of how EVs are involved in initial tissue destruction and, subsequent mechanisms of repair, are still unclear but research is working towards answering some of these questions and in doing so are finding that the EVs are playing an even more critical role than first anticipated. Indeed, it seems that if the EVs could be isolated in a reproducible and controlled manner and then profiled they would provide us with a vast amount of data that could be used to target OA detection and prevention. The unique bio-cargo of EVs make them prime candidates for novel biomarkers of disease progression and potentially, also determining if patients are pre-disposed to OA. The ability to understand how the EVs function within the joint would allow us to target certain pathways to prevent tissue destruction or enhance the tissues natural repair mechanisms. If we were to understand more fully the involvement of EVs in the mechanisms of OA, the future for these valuable biomolecules is promising. This could potentially lead to a huge surge in research towards cartilage repair therapeutics, an outcome that the millions of OA sufferers worldwide would truly appreciate.
